# Supportive development of functional tissues for biomedical research using the MINUSHEET® perfusion system

**DOI:** 10.1186/2001-1326-1-22

**Published:** 2012-10-05

**Authors:** Will W Minuth, Lucia Denk

**Affiliations:** 1 Department of Molecular and Cellular Anatomy, University of Regensburg, Regensburg, Germany

**Keywords:** Cell culture, Tissue culture, Tissue engineering, Biomaterial, MINUSHEET® tissue carrier, Perfusion culture, Gradient perfusion culture, Artificial polyester interstitium

## Abstract

Functional tissues generated under in vitro conditions are urgently needed in biomedical research. However, the engineering of tissues is rather difficult, since their development is influenced by numerous parameters. In consequence, a versatile culture system was developed to respond the unmet needs.

Optimal adhesion for cells in this system is reached by the selection of individual biomaterials. To protect cells during handling and culture, the biomaterial is mounted onto a MINUSHEET® tissue carrier. While adherence of cells takes place in the static environment of a 24 well culture plate, generation of tissues is accomplished in one of several available perfusion culture containers. In the basic version a continuous flow of always fresh culture medium is provided to the developing tissue. In a gradient perfusion culture container epithelia are exposed to different fluids at the luminal and basal sides. Another special container with a transparent lid and base enables microscopic visualization of ongoing tissue development. A further container exhibits a flexible silicone lid to apply force onto the developing tissue thereby mimicking mechanical load that is required for developing connective and muscular tissue. Finally, stem/progenitor cells are kept at the interface of an artificial polyester interstitium within a perfusion culture container offering for example an optimal environment for the spatial development of renal tubules.

The system presented here was evaluated by various research groups. As a result a variety of publications including most interesting applications were published. In the present paper these data were reviewed and analyzed. All of the results point out that the cell biological profile of engineered tissues can be strongly improved, when the introduced perfusion culture technique is applied in combination with specific biomaterials supporting primary adhesion of cells.

## Review

### Background

In tissue engineering, biomaterial research and regenerative medicine culture experiments with epithelial, connective, muscular and nervous tissues are of extreme importance. In the focus of interest are cellular interactions with innovative biomaterials, controlled tissue regeneration and toxic influences of newly developed pharmaceuticals preventing inflammation but promoting renewal of tissues.

While the expansion of isolated cells in the static environment of a culture dish is easy to perform, developing tissues frequently illustrate severe morphological, physiological and biochemical alterations caused by dedifferentiation especially in combination with innovative biomaterials [1-8]. Atypical features are not evoked by one parameter, but are influenced on the one hand by suboptimal material surfaces resulting in minor cell adhesion and communication. On the other hand culture conditions such as suboptimal nutrition, low respiratory gas or unstirred layers of fluid are responsible for the process of atypical development [9-14]. Consequently, several parameters have to complement one another for an optimal generation of tissues. Since a classical dish does not comply to necessary needs of cells, advanced culture techniques allowing creation of an adaptable environment have to be applied for intact development of tissues [15].

Due to the multiple parameters that influence tissue development a modular system is presented here that adjusts culture conditions to individual needs of developing tissues [16]. Since suitable equipment for tissue engineering was at that time not commercially available, the described tools were developed for the own laboratory work. The innovative concept is based on a MINUSHEET® tissue carrier, which enables the researcher to apply individually selected biomaterials promoting in turn optimal cell adhesion. After transfer of a tissue carrier in a compatible perfusion culture container, the fluid environment can be individually machted to the contained tissue. Thus, the modular system provides a highly adaptable basis for adhesion of cells in a MINUSHEET® tissue carrier with subsequent culture in the static environment of a 24 well culture plate. The compatible tissue carriers in combination with different kinds of perfusion culture containers made it possible to generate specialized tissues under continuous provision with always fresh medium.

### Modulating tissue development by an adapted environment

Under natural conditions a basic prerequisite for optimal tissue development is a positive interaction of adherent cells with the extracellular matrix. Under in vitro conditions a selected biomaterial has to replace the naturally occurring extracellular matrix.

#### Balance between differentiation and dedifferentiation

Tissue engineering experiments demonstrated that the composition of applied materials such as synthetic polymers, decellularized matrix, biodegradable scaffolds, ceramics or metals has a strong influence on the adhesion of cells and consequently on the generation of tissues [17-26]. Furthermore, the roughness of an applied biomaterial can determine the development of maturing tissue in a good or bad sense. Also the spatial contour of biomaterials available in form of filters, nets, foils, fleeces or foams including small or big pores plays an essential role in regulation of tissue formation. Regarding the huge amount of newly developed biomaterials the challenge for the future is to recognize as early as possible the respective positive influences or toxic features on adherent cells by an advanced culture system.

#### Mounting biomaterials in a tissue carrier

**Figure 1:**
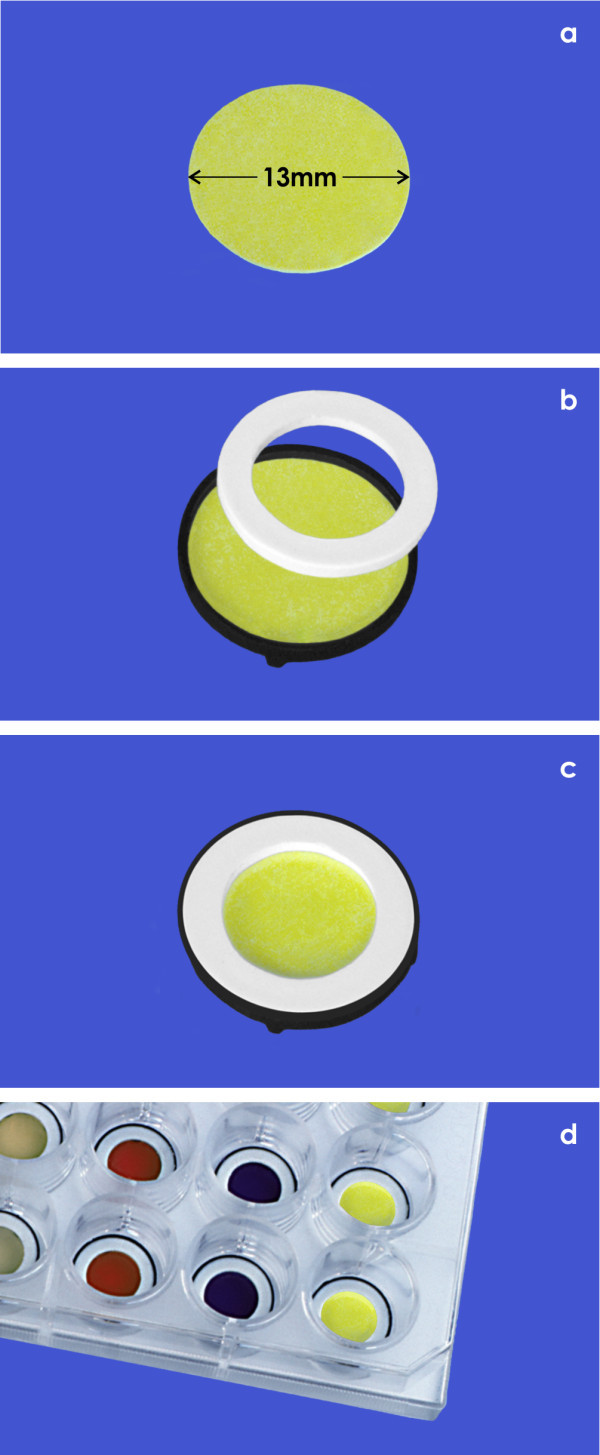
**Schematic illustration of mounting a MINUSHEET® tissue carrier.** (**a**) First an individual biomaterial supporting optimal tissue development is selected. (**b**) For mounting the biomaterial measuring 13 mm in diameter is placed in the black base part of a tissue carrier. (**c**) After fixation by a white tension ring the mounted tissue carrier can be used for cell seeding. For easy access of medium at the basal side a tissue carrier rests with protrusions on the bottom of a dish. (**d**) Tissue carriers including different biomaterials are demonstrated within a 24 well culture plate.

Adherent cells can be cultured on individually selected biomaterials as a substitute for the extracellular matrix. To stay compatible with a 24 well culture plate, the selected biomaterials are excised by a punching tool to a diameter of 13 mm or are ordered in this format (Figure 1a). For easy handling and to prevent damage during culture, the selected material is mounted in the base part of a MINUSHEET® tissue carrier (Figure 1b; black ring). The biomaterial is held in position by pressing it down with a tension ring (Figure 1c; white ring). The mounted tissue carrier is then enveloped and sterilized depending on the chemical composition of contained biomaterial either in formalin, ethylene oxide gas atmosphere or in an autoclave. Subsequently the tissue carrier can be frozen, stored at room temperature or used immediately for cell seeding.

#### Seeding of cells on a tissue carrier

For cell seeding the tissue carrier including the biomaterial of choice is placed by forceps in a 24 well culture plate (Figure 1d). To increase cell concentration, the selected biomaterial is solely wetted with culture medium. A standard culture on a MINUSHEET® tissue carrier can be performed by seeding cells onto the upper side of the selected biomaterial. When the carrier is turned, cells can be also seeded on the basal side so that co-culture with two different cell types becomes possible [27,28].

Cells are transferred to the selected biomaterial by a pipette within a small droplet of medium. Since the tissue carrier rests on small protrusions, cells are provided from the upper and lower side by medium. Culture is started with a conventional medium in a CO_2_ incubator until primary adhesion of cells is replaced by adherence. Depending on applied biomaterial and cell type this process can last a few hours or even days.

Regardless of whether transparent or non transparent biomaterials are used, the degree of cell adhesion can be easily registered by fluorescence microscopy after ethanol fixation and propidium iodide incubation [29]. Such a protocol illustrates fluorescent nuclei of cells adhering to a biomaterial. For example, when MDCK cells were cultured onto four different materials such as glass, polystyrene (Thermanox®), a white or black polycarbonate filter, it was observed that each of the specimens reflects an individual growth pattern of cells ranging between perfect confluent adherence and atypical dome, blister and cluster formation respectively. This result illustrates that selected biomaterials have an enormous influence on the spatial growth pattern of adherent cells.

Not only single cells but also a thin slice of tissue can be mounted between two pieces of a woven net or a fleece within a MINUSHEET® tissue carrier [30,31]. In addition, flexible collagen sheets for adhesion of cells can be stretched like the skin of a drum in a slightly modified carrier [32,33]. The few examples show that principally a broad spectrum of biomaterials can be inserted in a tissue carrier so that cells obtain an adequate surface for adhesion or adherence to develop further into a specialized tissue.

#### Compatible perfusion culture containers

**Figure 2:**
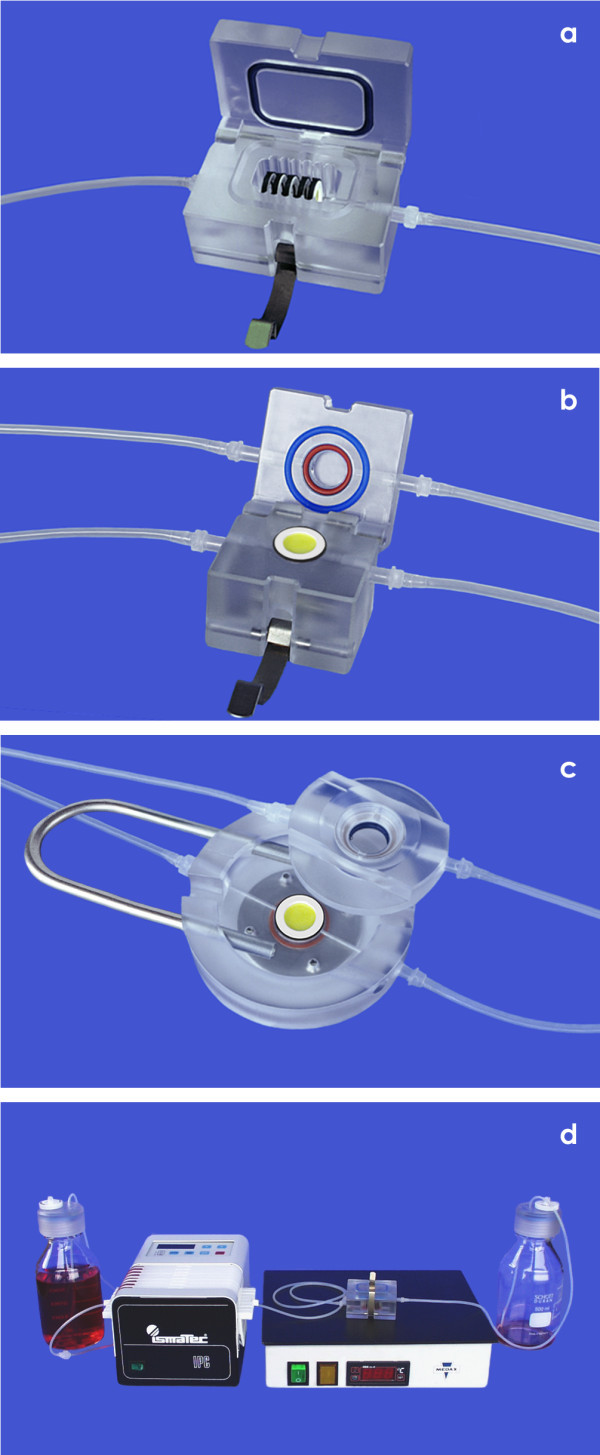
**Application of a Minusheet® tissue carrier in compatible perfusion culture containers.** (**a**) A first type of perfusion culture container can hold six tissue carriers to provide them with always fresh medium. (**b**) In a gradient perfusion culture container the respective tissue is exposed to different fluids at the luminal and basal side. (**c**) In a microscope container with a transparent lid and base developing tissue can be observed. (**d**) Perfusion culture set-up is running on a laboratory table. A thermo plate maintains the desired temperature of 37°C. During culture a peristaltic pump transports the medium (1.25 ml/h) from a storage bottle (left side) to the waste bottle (right side).

It is obvious that the static environment within a culture dish leads to an uncontrollable increase of metabolites and a decrease of nutrition during time. Due to this reason a MINUSHEET® tissue carrier with adherent cells is used only for the relatively short period of cell seeding in the static environment of a 24 well culture plate. To provide constant nutrition and respiratory gas during further tissue development, the tissue carrier is consequently inserted in a perfusion culture container. The exact geometrical placement of the tissue carrier within a perfusion culture container guarantees on the one hand an equal distribution and on the other hand a constant provision with always fresh culture medium. The basic version of a container allows continuous medium transport to developing tissues from all sides (Figure 2a). In a gradient container the tissue carrier is held between the base and the lid so that the luminal and basal sides can be provided with individual media mimicking a typical environment for epithelia (Figure 2b). A further perfusion container is made of a transparent lid and base allowing the microscopic observation during tissue development (Figure 2c). Yet another specific container exhibits a flexible silicone lid. Applying force to this lid by an eccentric rotor simulates a mechanical load, which is required in cartilage and bone tissue engineering. Shaped tissues such as an auricle or different forms of cartilage implants can be generated with individual scaffolds in a special tissue engineering container. Finally, spatial extension of tubules derived from renal stem/progenitor cells is obtained within a perfusion culture container filled with an artificial interstitium consisting of a polyester fleece [30,31]. 

In conclusion, compared to other perfusion culture experiments the presently described system exhibits up to date unique features for the generation of specialized tissues. Damage during culture and transfer of growing tissue is prevented by applied MINUSHEET® tissue carriers (Figure 1). Adhesion of cells to natural extracellular matrix or synthetic biomaterials can be modulated by mounting individual supports (Figure 1a-c). While seeding of cells and initial culture can be performed in the static environment of a 24 well culture plate (Figure 1d), the exact positioning of a carrier within a perfusion container makes it possible to provide developing tissue with always fresh medium (Figure 2a). It prevents an overshoot of paracrine factors and keeps concentration of harmful metabolites low. Finally, offering different media at the luminal and basal side within a gradient culture container allows simulation of a natural barrier environment for epithelia (Figure 2b). Thus, the described technique bridges consequently static culture in a conventional 24 well plate (Figure 1d) with modern perfusion culture in different perfusion culture containers (Figure 2).

#### Permanent supply of fresh culture medium

To maintain the desired temperature of 37°C within a perfusion culture container a heating plate (MEDAX-Nagel, Kiel, Germany) and a cover lid (not shown) is used (Figure 2d). The transport of culture medium is best accomplished using a slowly rotating peristaltic pump (ISMATEC, IPC N8, Wertheim, Germany), which is able to provide adjustable pump rates of 0.1 to 5 ml per hour. In personal experiments medium was transported with 1.25 ml/h for a period of at least 13 days. Adherent cells growing on a mounted tissue carrier are supplied with always fresh medium flowing from the storage bottle to the container. Thus, during long term culture the growing tissue is exposed to always fresh medium preventing in parallel an un-physiological accumulation of metabolic products and an overshoot of paracrine factors. To maintain this controlled environment for the whole culture period, the metabolized medium is not re-circulated but collected in a separate waste bottle.

#### Keeping the pH constant in culture medium

Working with perfusion culture equipment under atmospheric air on a laboratory table facilitates the complete handling. However, in the majority of cases conventional cell and tissue cultures are performed in a CO_2_ incubator. In this case the applied media are buffered by a system consisting of a relatively high amount of NaHCO_3_ and a 5% CO_2_ atmosphere to maintain a constant pH between 7.2 and 7.4. If such a formulated medium is used for perfusion culture outside a CO_2_ incubator, the pH will shift from the physiological range to much more alkaline values due to the low content of CO_2_ (0,3%) in atmospheric air.

For that reason any medium used for perfusion culture outside a CO_2_ incubator has to be stabilized by reducing the NaHCO_3_ concentration and/or by adding biological buffers such as HEPES (GIBCO/Invitrogen, Karlsruhe, Germany) or BUFFER ALL (Sigma-Aldrich-Chemie, München, Germany). The necessary amount can be determined by admixing increasing amounts of biological buffer solution to an aliquot of medium. Then the medium must equilibrate over night on a thermo plate at 37°C under atmospheric air. For example, application of 50 mmol/l HEPES or an equivalent of BUFFER ALL (ca. 1%) to IMDM (Iscove’s Modified Dulbecco’s Medium, GIBCO/Invitrogen) maintains a constant pH of 7.4 throughout long term perfusion culture under atmospheric air on a laboratory table.

#### Oxygen content in transported culture medium

When a perfusion culture experiment is performed, the pH must be adjusted and sufficient respiratory gas must be present. To enrich oxygen (O_2_) for example in IMDM, the medium has to be transported through a gas-permeable silicone tube. Such a silicone tube provides a large surface for gas exchange by diffusion due to a thin wall (1 mm), the small inner diameter (1 mm) and its extended length (1 m). Analysis of IMDM (3024 mg/l NaHCO_3_, 50 mmol/l HEPES) equilibrated against atmospheric air during a standard perfusion culture experiment consequently showed partial pressures of 160 mmHg O_2_ and 12 mmHg CO_2_[34].

Furthermore it has to be considered that depending on the tissue types the requirements of oxygen are individual. Therefore it is important to note that the content of oxygen can be adapted in individual perfusion culture set-ups. The technical solution is a gas exchanger module containing a gas inlet and outlet. Moreover a spiral with a long thin-walled silicone tube for medium transport is mounted inside the module. Since the tube is highly gas-permeable, optimal diffusion of gases between culture medium and surrounding atmosphere within the gas exchange module is guaranteed. In consequence, the desired gas atmosphere can be adjusted by a constant flow of a specific gas mixture through the module. That way the content of oxygen or any other gases can be modulated in the medium by diffusion. By applying this simple protocol it became possible to decrease the oxygen partial pressure within the transported medium during long term culture under absolutely sterile conditions [34]. These kinds of experiments elicited that a decrease in oxygen concentration was leading to a massive alteration of functional protein expression in generated renal collecting duct epithelia.

#### Elimination of harmful gas bubbles

During perfusion culture experiments gas bubbles can arise and influence in turn the flow of medium. Formation of gas bubbles is not only observed during suction of medium from the storage bottle but also during transport at material transitions between tubes and connectors.

First, these gas bubbles are so small that they cannot be recognized, but during ongoing transport of culture medium they increase in size and are able to form an embolus that massively impedes medium flow. Second, gas bubbles can accumulate in the culture container leading to a regional shortage of medium supply and breaks in the fluid continuum so that remarkable fluid pressure changes result. Most importantly, in a gradient perfusion culture experiment, in which two media are transported at exactly the same speed embolic effects can lead to pressure differences, which in turn destroy the barrier function of the contained epithelia [35].

To minimize the content of gas bubbles within a perfusion culture set-up, a gas expander module was developed [36]. This module removes gas bubbles from the medium. When medium is entering the module, it rises within a small reservoir and expands before it drops down onto a barrier. During this process gas bubbles are separated from the medium and collected at the top of the gas expander module. As a result, medium leaving the gas expander module is oxygen-saturated but free of gas bubbles.

### Wide spectrum of applications

When tissues are generated in combination with new biomaterials, it is expected that they develop a high degree of functionality similar to the one known from the respective organism. Such a tissue-specific quality cannot be obtained by the use of a conventional dish, but is reached by offering an optimal surface for cell adhesion, compatible perfusion culture containers, permanent transport of nutrition including respiratory gas and constant elimination of metabolic products. Years ago such a compatible system was not available on the market. In consequence, necessary tools such as the previously described tissue carriers and versatile perfusion culture containers were developed for performance of culture experiments in the own laboratory (Figure 1 and 2).

After publication of successful results also other groups were interested in application of the innovative technique. As a result numerous papers were published dealing with the MINUSHEET® perfusion culture technique. A list of those different culture set-ups is found in the data bank ‘Proceedings in perfusion culture’: http://www.biologie.uni-regensburg.de/Anatomie/Minuth/proceedings.htm

### Creating an improved fluid environment for epithelia

Experiments have shown that epithelial cells often do not develop expected cell biological features when they are cultured at the bottom of a conventional dish. Consequently, MINUSHEET® tissue carriers including an optimal biomaterial and compatible perfusion culture containers were applied to offer an improved environment.

#### Keeping specialized epithelia in perfusion culture

Pilot experiments were made with collecting duct (CD) cells derived from the embryonic parenchyma of neonatal kidney [37,38]. By applying the mentioned tissue carriers and perfusion culture containers polarized epithelia were harvested for the first time expressing under in vitro conditions cell biological features such as observed in adult Principal (P) and Intercalated Cells (IC) of the renal collecting duct [33,39-42]. In the following experiments special attention was directed to the selection of individual biomaterials improving adhesion of cells so that they can stand fluid flow in perfusion culture [29]. Further experiments with cells derived from embryonic parenchyma brought new insights into the spatial development of renal microvasculature and glomeruli [43-46]. In addition, perfusion culture was performed to accelerate regeneration by engineered microvessels [47,48] and to investigate restoration of endothelium [49,50].

To bridge time before an implantation is made, perspectives of living conservation of human gingival epithelium was investigated in long term perfusion culture experiments [51,52]. Factors affecting reproductive aging and the development of fertilized eggs were investigated with anterior pituitary gland [53], oviduct epithelium [54] and endometrium [55]. Optimal matrix coating and adaption of continuous medium flow were elaborated for hepatocytes [56-59]. Regeneration of urothelium was investigated in combination with newly developed stent materials [60]. Effects of newly developed pharmaceuticals on ciliary beat frequency (CBF) were elaborated by differentiated nasal epithelium generated in described perfusion culture [61].

#### Exposure of epithelia to a luminal-basal fluid gradient

When performing perfusion culture experiments one has to consider that the environment of epithelia is changing during development. In the fetal period epithelia are exposed to the same fluid composition at the luminal and basal sides due to the still leaky barrier. However, in maturing epithelia a tight junction complex and up-regulated transport features are leading to a functional barrier. Because of the physiological seal different media are found at the luminal and basal sides [15].

When epithelial cells are cultured at the bottom of a conventional dish, all sides are exposed to the same medium. This untypical situation is leading to a permanent biological short circuit promoting cell proliferation but suppressing polar differentiation [62-67]. To overcome this problem, MINUSHEET® tissue carriers can be mounted in a gradient perfusion culture container to mimic at the luminal and basal sides a tissue-specific environment for epithelia. Following this strategy finally functional renal membranes were generated [68,69].

**Figure 3:**
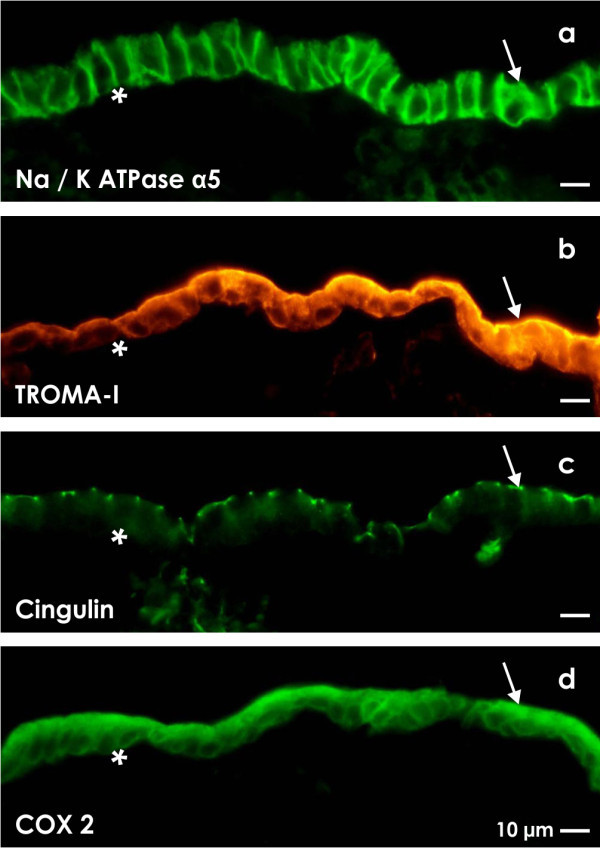
**Cell biological features of an embryonic renal collecting duct (CD) epithelium kept for 13 days in a fluid gradient.** In the gradient container at the luminal side IMDM + aldosterone (1 × 10^-7^ M) + 15 mmol/l NaCl, while at the basal side IMDM + aldosterone (1 × 10^-7^ M) was transported. Immunohistochemistry shows a positive label for tissue-specific (**a**) Na/K ATPase α5, (**b**) TROMA I, (**c**) cingulin and (**d**) COX 2 protein. In contrast, perfusion culture of epithelia with IMDM at the luminal and basal side does not develop these features. Basal lamina (asterisk), lumen (arrow).

Originally experiments with a gradient perfusion culture container were performed to investigate the influence of different fluids at the luminal and basal sides of embryonic renal collecting duct (CD) epithelia [32,33,38,70-76]. These experiments showed that the development of a CD epithelium has an unexpected long latent period of three days. Moreover it takes at least 10 days until typical features of differentiation are up-regulated. The development is also depending on increasing NaCl concentrations specifically offered at the luminal side (Figure 3). Surprisingly, in a continuous electrolyte gradient over many days typical epithelial features such as binding of lectins or expression of site-specific proteins such as Na/K ATPase α5 (Figure 3a), TROMA I (Cytokeratin Endo-A; Figure 3b), cingulin (Figure 3c) and COX 2 (Figure 3d) were found to be up-regulated. However, when the increased NaCl content at the luminal side was replaced against a low NaCl concentration, gained features were found to be down-regulated. This result illustrates that the luminal-basal electrolyte gradient maintains functional features within renal epithelia. 

Further challenging experiments were performed with hydrogel in MINUSHEET® tissue carriers mimicking the glomerular basement membrane. In this experimental set-up it was possible to seed endothelial cells on the one side and podocytes on the other side. Mounting these co-cultures in a gradient perfusion container, development of urine-blood barrier functions were tested [77]. In so far gradient perfusion culture made it possible to keep renal epithelia in vitro as observed under natural conditions. Furthermore such a tissue-specific development made it apparent that the applied media had to be adapted to the physiological requirements by addition of defined electrolytes [34,78,79].

#### Maintenance of retina

It has been shown that the retina is a complex tissue lined by a pigment epithelium that cannot be maintained in the static environment of a culture dish over prolonged periods of time. To improve the environment, intact retina was mounted onto a tissue carrier for culture within a gradient perfusion container [80-86]. It was demonstrated that pigment epithelium and neighboring neurons maintain a perfect morphology for at least 10 days. These exiting findings illustrate innovative perspectives for safety testing of newly developed pharmaceuticals designed for the intraocular medical application. Furthermore these experiments show challenging options to investigate the wide field of retina aging, degeneration and repair under realistic culture conditions [87-91].

#### Blood-retina and blood-brain barrier in perfusion culture

Both blood-retina and blood-brain barrier are crucial for the transport of pharmaceuticals. Gradient perfusion culture appears as an ideal technique to simulate such a blood-retina and blood-brain barrier under realistic in vitro conditions [92-94]. Indeed, the perfusion culture experiments showed new features of permeation and displayed an intact polarized expression of efflux pumps such as multidrug resistance protein (P-gp) and multidrug resistance-associated protein (MRP).

#### Creating a blood-air barrier

A specific environment for pneumocytes in form of a blood-air barrier was simulated in gradient perfusion culture [28]. When pneumocytes and endothelial cells were co-cultured on a polycarbonate filter within a gradient perfusion container, development of a tight junction complex was observed sealing perfectly the blood-air barrier. Further characteristic features of polar differentiation within the epithelia were found to be up-regulated. It was argued that gradient perfusion culture in combination with pneumocytes and endothelial cells is a realistic model to investigate dose-controlled exposure of airborne particles. Moreover, to elucidate barrier transport and repair mechanisms after alveolar injury a dose controlled air-liquid interface (ALI) was investigated in a gradient perfusion container by using A549 cells [95-97].

#### Constructing a blood-gas barrier

Culture experiments on fish swim bladder gas gland were successfully performed in a gradient perfusion container [98]. For these series of experiments cells of gas gland were cultured on a filter at the interface between gas and culture medium. The generated epithelium revealed a typical polarity and functionality as it was observed in the environment of swim bladder gas gland in the fish.

#### Pharmaceutical applications applying a gradient container

Most of the administered pharmaceuticals have to pass an epithelial barrier in the organism. To test the transport of newly developed drugs through an epithelial cell layer long term gradient perfusion culture experiments were performed [99,100]. In these series of experiments it was found that Caco-2 cells are forming a tightly sealing epithelial cell layer [101,102]. Further gradient perfusion culture exhibited that reproducible results can be achieved much earlier than observed in traditional 21 day static cultures. Interestingly the permeability coefficient of several model pharmaceuticals across a Caco-2 cell layer was approximately twofold higher than obtained under conventional static conditions.

#### Renewal of skin

The regeneration and repair of skin is an important research area in actual biomedicine. Consequently, epidermis equivalents were generated by using gradient perfusion culture [103]. In this investigation composite grafts of INTEGRA® matrix and human keratinocytes were cultured in a gradient container in order to evaluate the potential of the cost-effective engineering of full-thickness skin grafts and the treatment of ulcers. Furthermore it was demonstrated that generation of gingival epithelium [52,104] or co-culture of keratinocytes with osteoblast-like cells kept in perfusion culture results in a much better tissue generation than observed under static culture conditions [105].

### Offering an artificial interstitium for regenerating parenchyma

**Figure 4:**
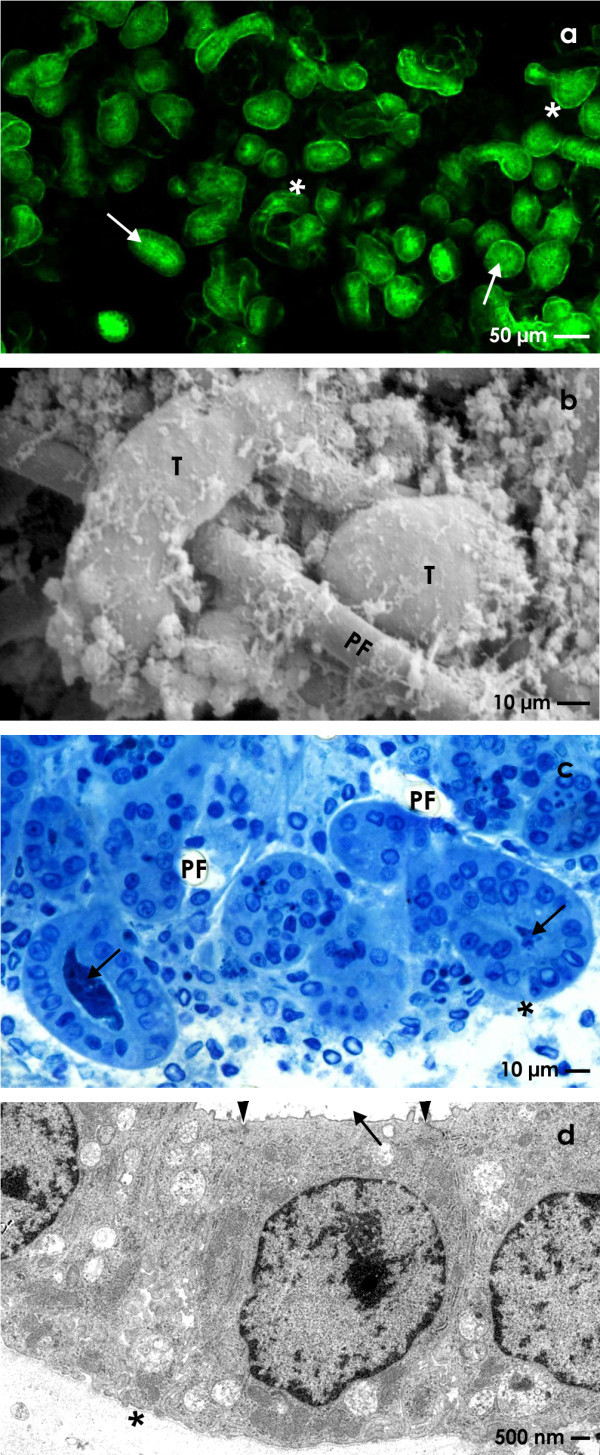
**Generation of renal tubules at the interface of an artificial interstitium.** (**a**) SBA label visualizes in an individual sample tubules developing within an artificial interstitium consisting of a polyester fleece. (**b**) Scanning electron microscopy illuminates growth of renal tubules (T) between fibers of the polyester fleece (PF). (**c**) Semithin section after Richardson staining shows generated tubules in oblique and vertical view. (**d**) Transmission electron microscopy demonstrates that regenerating tubules contain a polarized epithelium. Neighboring cells are separated by a tight junctional complex (arrow head). Basal lamina (asterisk), lumen (arrow).

The repair of renal parenchyma is of special interest in acute and chronic kidney failure. To investigate the spatial regeneration of renal tubules under realistic conditions, an advanced MINUSHEET® perfusion culture protocol was developed (Figure 4). To replace coating by extracellular matrix proteins renal stem/progenitor cells were mounted between layers of a polyester fleece. The interface between the polyester fibers creates an artificial interstitium promoting spatial extension of tubules [106,107]. For example, SBA lectin labeling of a specimen kept for 13 days in perfusion culture illustrates that numerous tubules can be detected in this specific area (Figure 4a). Each of the tubules exhibits a lumen and a basal lamina. Scanning electron microscopy further illuminates the spatial growth of generated tubules between polyester fibers (Figure 4b). Semithin sections (Figure 4c) and transmission electron microscopy (Figure 4d) clearly demonstrate that generated tubules contain a polarized epithelium. In the meantime numerous data demonstrated that perfusion culture together with an artificial interstitium is an effective technique to investigate the process of tubule regeneration [108-110]. In addition, for the first time it was shown that tubule formation can be induced by aldosterone, while antagonists such as spironolactone or canrenoate prevent tubulogenic development [111-113]. By preventing the molecular contact between the mineralocorticoid receptor (MR) and heat shock protein 90 geldanamycin leads to a lack of tubule formation. In consequence, the results demonstrate that the signal initiated by aldosterone is not unspecific but is mediated via MR and depends on intact molecular interactions with heat shock proteins. 

It is a new aspect that the interface between layers of polyester fleece promotes the spatial development of numerous tubules [114,115]. When such an artificial interstitium is applied, the surrounding of generating tubules is not stacked by coated extracellular matrix proteins. In consequence, it became possible for the first time to investigate the spatial growth of tubules by scanning electron microscopy and the synthesis of special interstitial molecules during regeneration (Figure 4b). Recent data further illustrate that developing tubules avoid a direct contact to each other by keeping a discrete distance during spatial development. The separation seems to be caused by an interaction between the basal lamina of generating tubules, newly synthesized fibers of the extracellular matrix and neighboring polyester fibers of the fleece [116-122].

Finally, perfusion culture experiments exhibited that application of different kinds of polyester fleeces are leading to various patterns of spatial tubule development resulting in new challenging perspectives for the regeneration of diseased renal parenchyma [31]. By performing these pilot experiments it became evident that aldosterone has a stimulating influence on the formation of renal tubules, while glucocorticoids are inducing atypical structures in form of extended cell clusters [122]. In conclusion, it is an important new finding that steroid hormones occurring in the interstitial fluid may exhibit a harmful influence on the regeneration of renal parenchyma.

### Engineering of connective tissue in perfusion culture

A big research field for tissue engineering and biomaterial research is the generation of connective tissue constructs. In this coherence it is barely considered that beside the epithelial cell layers also connective tissue can build up important barrier functions.

#### Connective tissue barrier

For example, experiments related to such a non-epithelial barrier were performed with dentin discs mounted in a MINUSHEET® gradient perfusion container [123-132]. Most interestingly, these data reveal that polymerized dental resin materials release residual monomers that may interact with pulp tissue. In consequence, to obtain information about diffusion of molecules through a dentin disc gradient perfusion culture appears to be an ideal model to investigate long term toxic effects under realistic in vitro conditions. Finally, new aspects of testing permeability and degradation in gelatine membranes were obtained by keeping fibroblasts in a gradient perfusion container [133].

#### Generation of hyaline cartilage

A great challenge in tissue engineering is the treatment of cartilage defects by regenerating chondrocytes growing onto innovative scaffold materials. In numerous cases it was demonstrated that MINUSHEET® perfusion culture can improve the cell biological quality of generated cartilage. An enormous advantage of the described technique is that damage of developing tissue within the scaffold is minimized due to the permanent elimination of biodegraded molecules by transported culture medium. For example, perfusion culture was applied successfully for the controlled regeneration of hyaline cartilage [18,21,134,135]. In these experiments it became possible to elaborate exact data concerning kinetics of the degradation process from different scaffold materials [136,137]. Furthermore the cell biological quality of generated cartilage was improved by stepwise chemical modifications of the scaffold material by perfusion culture. Following this strategy the risk of tissue repulsion after implantation could be decreased by selecting optimal scaffold materials [138-145]. Surprisingly, it was demonstrated that the application of natural extracellular matrix such as a collagen sponge does not improve the quality of generated cartilage [146]. In contrast, scaffold materials with modified polyethylene coating [147] or a gelatine-based Spongostan® [23] revealed much more cartilage specific features than observed without surface treatment. It was further detected that synovial fibroblasts are able to modulate articular matrix synthesis [148]. Finally, tissue engineering of cartilage constructs by perfusion culture seemed to be an ideal model to investigate parameters affecting destructive joint diseases [149-151].

#### Formation of bone constructs

Beside cartilage formation MINUSHEET® perfusion culture technique was also applied for bone tissue engineering to investigate the development of osteoblasts on ceramic materials [24-26,152,153], decellularized spongeous bone [154], collagen membranes [155], mineralized collagen [156,157], hydroxyapatite scaffolds [158-161], PLGA sheets [162], iron based metals [163], bioactive glass [164], textile chitosan [165-167] or 3D biphasic calcium phosphate [168] scaffolds and biocorrodible bone replacement materials [169]. Finally, most important for clinical applications are experiments, which exhibit that bone development can be influenced by the sterilization procedure of scaffolds consisting of poly-d,l-lactic-co-glycolic acid [162].

A permanently occurring problem in bone tissue engineering is that unstirred and consequently harmful layers of fluid within growing tissue can arise. To compensate for this the continuous provision with nutrition and oxygen must be replaced by transportation of fluid in pulses so that generation of bone constructs results in an increased cell biological quality [170]. Last but not the least, the insights gained by bone formation may lead to an effective strategy for the regeneration of dentin [127].

### Growth of muscular tissue

Surprisingly, only three papers were found that deal with the regeneration of muscular tissue in MINUSHEET® perfusion culture. When gastric mucosa was kept in a culture container, it was recognized that also smooth muscular tissue is developing within the lamina propria [67]. By applying improved biomaterials for cell seeding numerous cerebral pericytes were found to express site-specific pericytic aminopeptidase N/pAPN [171]. Proliferation and adhesion of smooth muscle cells was investigated on electrospun polymer scaffolds [172].

### Development of nervous tissue

One of the main subjects in the area of neurology is the escape of dopamine synthesis during Parkinson’s disease. To investigate influences affecting synthesis of dopamine, MINUSHEET® perfusion culture was performed successfully using mesencephalic neurons [173]. These experiments demonstrated for example that neurothrophins stimulate the release of dopamine via Trk and p75Lntr receptors. Furthermore it was shown in hippocampal neurons and the pheochromacytoma cell line PC 12 that application of exogenous neurotrophins exhibits positive feedback effects on secretion of synthesized neutrotrophins. This pathway is mediated via an activation of tyrosine kinase neurotrophin receptors [174]. The influence of sodium in an activity-dependent secretion of neurotrophins plays thereby an important role [175]. Further significant differences in the secretion between nerve growth factor and brain-derived neurotrophic factor were observed [176]. Finally, SH-SY5Y human neuroblastoma cells were found to differentiate into a neuronal-like state in long term perfusion culture [177]. The cells could be maintained in an active state for more than two months without the need of passaging them. In a further set of experiments RAT-1 fibroblasts were investigated expressing Cypridina noctiluca luciferase (CLuc) driven by the promoter of the circadian clock gene Mma11 [178]. The performed experiments showed that the CLuc reporter assay in combination with the described perfusion culture appears to be an innovative pharmacological tool for drug discovery. Finally, most promising results were obtained with fish pituitary explants to investigate vasotocin and isotocin release when kept in perfusion culture [179].

## Conclusions

MINUSHEET® perfusion culture technique was developed to improve the environment of adherent cells and developing tissues. For optimal adhesion of cells an individual biomaterial is selected and mounted onto a tissue carrier. Seeding of cells is performed in the static environment of a conventional 24 well culture plate, while perfusion culture is performed with compatible containers offering an adequate fluid environment for the generation of specialized tissues. Numerous papers illustrate that the modular system is generating cells and tissues in excellent cell biological quality urgently needed in tissue engineering, biomaterial research and advanced pharmaceutical drug testing.

### Final remarks

Numerous patents (not listed) demonstrate that Will W. Minuth has invented the presented MINUSHEET® perfusion culture technique. To introduce the developed tools on the market Katharina Lorenz-Minuth founded non-profit orientated Minucells and Minutissue GmbH (D-93077 Bad Abbach/Germany, http://www.minucells.com). In 1992 the project received the Philip Morris research award ‘Challenge of the Future’ in München/Germany.

## Competing interests

The authors declare that they have no competing interests.

## Authors’ contributions

WM contributed to the collection of information, interpretation of data and writing of the manuscript. LD performed the generation of renal tubules, histochemistry, electron microscopy, design of figures and critical revision of the manuscript. All authors read and approved the final manuscript.
